# Two Opsin 3-Related Proteins in the Chicken Retina and Brain: A TMT-Type Opsin 3 Is a Blue-Light Sensor in Retinal Horizontal Cells, Hypothalamus, and Cerebellum

**DOI:** 10.1371/journal.pone.0163925

**Published:** 2016-11-18

**Authors:** Mutsuko Kato, Takashi Sugiyama, Kazumi Sakai, Takahiro Yamashita, Hirofumi Fujita, Keita Sato, Sayuri Tomonari, Yoshinori Shichida, Hideyo Ohuchi

**Affiliations:** 1 Department of Cytology and Histology, Okayama University Graduate School of Medicine, Dentistry and Pharmaceutical Sciences, Okayama, Japan; 2 R&D Group, Olympus Corporation, Tokyo, Japan; 3 Department of Biophysics, Graduate School of Science, Kyoto University, Kyoto, Japan; 4 Department of Life Systems, Institute of Technology and Science, Tokushima University Graduate School, Tokushima, Japan; Dalhousie University, CANADA

## Abstract

*Opsin* family genes encode G protein-coupled seven-transmembrane proteins that bind a retinaldehyde chromophore in photoreception. Here, we sought potential as yet undescribed avian retinal photoreceptors, focusing on Opsin 3 homologs in the chicken. We found two Opsin 3-related genes in the chicken genome: one corresponding to encephalopsin/panopsin (Opn3) in mammals, and the other belonging to the teleost multiple tissue opsin (TMT) 2 group. Bioluminescence imaging and G protein activation assays demonstrated that the chicken TMT opsin (cTMT) functions as a blue light sensor when forced-expressed in mammalian cultured cells. We did not detect evidence of light sensitivity for the chicken Opn3 (cOpn3). *In situ* hybridization demonstrated expression of *cTMT* in subsets of differentiating cells in the inner retina and, as development progressed, predominant localization to retinal horizontal cells (HCs). Immunohistochemistry (IHC) revealed cTMT in HCs as well as in small numbers of cells in the ganglion and inner nuclear layers of the post-hatch chicken retina. In contrast, cOpn3-IR cells were found in distinct subsets of cells in the inner nuclear layer. cTMT-IR cells were also found in subsets of cells in the hypothalamus. Finally, we found differential distribution of cOpn3 and cTMT proteins in specific cells of the cerebellum. The present results suggest that a novel TMT-type opsin 3 may function as a photoreceptor in the chicken retina and brain.

## Introduction

Opsins are a family of membrane-bound, heptahelical G protein-coupled receptors characterized by their ability to bind retinaldehyde chromophores covalently via a Schiff base linkage [1]. There are seven major opsin subfamilies in chordates: melanopsin (opsin 4); encephalopsin/panopsin and teleost multiple tissue (TMT) opsin (opsin 3); ciliary photoreceptor opsins including rod/cone opsins, pinopsin, and vertebrate-ancient (VA) opsin; Go-coupled opsins; opsin 5 (formerly neuropsin); peropsin; and photoisomerases. Melanopsin is a non-canonical opsin expressed in the inner retina that mediates non-image forming effects of light on physiology, such as circadian photoentrainment [2]. Meanwhile, opsin 5, an ultraviolet light sensor expressed in the retina, was found to be expressed in the light-sensitive paraventricular organ of the avian hypothalamus as well, where it is involved in sensing day length and, consequently, modulating the size of sex organs in male birds across seasons [3–5].

Encephalopsin and TMT opsin, which belong to the opsin 3 subfamily, were originally discovered by database searches and low-stringency library screening; they have been observed to be expressed in the brain as well as in multiple other tissues in humans, mice, and teleosts (zebrafish and Fugu) [6–8]. Phylogenetic analyses have suggested that opsin 3 proteins are closely related to vertebrate photoreceptor opsins including rod/cone opsins, pinopsin, and vertebrate-ancient (VA) opsin [1,8,9]. TMT is expressed in hypothalamic neuro-sensory cells in teleosts and has been implicated in peripheral photoentraining in teleosts [8,10], suggesting that neuroendocrine photoregulation in vertebrates may involve TMT [9,11]. However, the expression patterns of *opsin 3* genes in the avian retina and brain have not been examined.

The aim of the present study was to explore whether there are as yet unidentified potentially photoreceptive cells in the avian retina and brain. We isolated two opsin 3-related genes expressed in the chicken retina, examined their photosensitivity, and analyzed their expression patterns in the chicken retina and brain.

## Materials and Methods

### Animals and ethics statement

Fertilized chicken eggs (*Gallus gallus*) were purchased from a commercial farm (Goto-furanjyo Co., Ltd.; Gifu, Japan; http://www.gotonohiyoko.co.jp/) and incubated at 37.5°C in a humidified incubator until they reached predetermined experimental time points. Hatchlings were housed in a 12:12 light-dark cycle with food and water *ad libitum*. Animals were anesthetized with ether and perfusion fixation combined tissue fixation with euthanasia at 6–10 h after lights on. The use of animals in these experiments was in accordance with the guidelines established by the Ministry of Education, Culture, Sports, Science, and Technology, Japan. The protocol was approved by the Committee on the Ethics of Animal Experiments of Okayama University (Permit Number: OKU-2013171).

### Isolation of chicken opsin 3-related genes

Partial cDNA fragments for two *opsin 3*-related genes were obtained by reverse transcriptase polymerase chain reaction (RT-PCR) from embryonic day 17 (E17) retina with primers designed according to predicted nucleotide sequences (S1 Table). Gene-specific primers were designed according to sequences deposited in GenBank (S1 Table). In total, 35 PCR cycles were performed at an annealing temperature of 58°C, generating PCR products of cDNA fragments (761 bp, 656 bp) similar to two opsin 3-related genes. To obtain full coding sequences for these genes, we first performed PCR with primers targeting regions containing translation initiation sites or stop codons, according to the nucleotide database, and obtained a full coding sequence for chicken encephalopsin, which we named chicken opsin 3 (cOpn3). For the other gene, which resembled the gene encoding TMT, we performed 3’RACE with cDNA from P7 retina and isolated long (cTMT-L) and short (cTMT-S) forms (S2 Table, S1 Fig). The deduced amino acid sequence of cTMT-S was identical to a sequence deposited in the Ensembl database (ENSGALG00000016802).

To obtain full-length cDNAs, RT-PCR was performed (primers shown in S1 Table). The PCR products were cloned into plasmid vectors [pGEM-T Easy (Promega; Madison, WI) or pCR4Blunt-TOPO (Invitrogen; Carlsbad, CA)] and multiple clones were sequenced to confirm that the entire coding cDNAs were obtained; *cOpn3* (1228 bp), *cTMT-S* (992 bp), and *cTMT-L* (1273 bp). The nucleotide sequence of these clones has been deposited in DDBJ/GenBank (accession numbers, AB436160, AB436159, and AB519059, respectively).

### Sequence and phylogenetic analysis

Assembly of predicted sequences, sequence analysis, and identity comparisons were undertaken in GENETYX-SV/RC Version 15 (Genetyx Co., Ltd.; Tokyo, Japan; https://www.genetyx.co.jp/). For phylogenetic purposes, amino acid sequences were aligned with MAFFT [12]; neighbor-joining trees were constructed with bootstrap confidence values based on 1000 replicates in MEGA7 software [13].

### Bioluminescence imaging

Bioluminescence assays were conducted with a Ca^2+^ indicator, cpGL-CaM [14], which contains a calmodulin-M13 Ca^2+^ sensor domain fused to a firefly luciferase [15] rendering its bioluminescent activity dependent on Ca^2+^ concentration. Neuro2A cells (ATCC, http://www.atcc.org/) were cultured in Dulbecco’s Modified Eagle Medium (Invitrogen) supplemented with 10% fetal bovine serum, 50 U/mL penicillin, and 50 μg/mL streptomycin. To enable transient expression, cells were plated on a 35-mm glass-bottom dish (Iwaki Co., Ltd.; Chiba, Japan; http://www.iwakipumps.jp/en), transfected with both cTMT-L and cpGL-CaM expression vectors (1 μg of each) facilitated by FuGENE HD transfection reagent (Promega), and incubated at 37°C for 24–48 h in the medium. Transfected Neuro2a cells were treated with 11-cis-retinal (5 μM) in the medium for 60 min. This and following procedures were performed in the dark. After retinal treatment, the cells were rinsed twice with basal salt solution (130 mM NaCl, 5.4 mM KCl, 2 mM CaCl_2_, 1 mM MgCl_2_, 10 mM D-glucose, and 10 mM HEPES, pH 7.4), and then soaked in basal salt solution supplemented with 2 mM D-luciferin. The cells were allowed to stabilize for 30 min prior to being subjected to imaging experiments.

For the experiments, a culture dish was placed on a microscopic luminescence imaging system (Lumino View LV200; Olympus; Tokyo, Japan) stage. Cells were observed with a 40× objective and maintained at room temperature (~24°C) throughout the experiments. Stimulating light from a 100-W halogen bulb was passed through a 470- to 490-nm or 535- to 555-nm band-pass filter and guided to illuminate the microscope stage. Optical power on the stage was measured by an optical power meter (Advantest Co., Ltd.; Tokyo, Japan; https://www.advantest.com/). Bioluminescence images (acquired immediately before and after, but not during light pulses to protect the camera from light overload) were collected every 10 s by a cooled electron multiplying charge-coupled device camera (iXon; Andor Technology Ltd.; Belfast, UK).

The resultant images were analyzed in MetaMorph software. Each cell was chosen as a region of interest (ROI), and its luminescence intensity was measured at experimentally specified time points.

### G protein activation assay

The cTMT-L action spectrum was obtained by plotting G protein activation efficiency of cTMT-L recombinant proteins, prepared as described previously [16], as a function of wavelength of light. The cTMT-L cDNA was inserted into pMT4 mammalian expression vector and transfected into HEK293T cells. After 1 day of incubation, 11-*cis* retinal was added to the medium (final concentration, 5 μM). After an additional 1-day incubation in the dark, the cells were collected. Collected cells were suspended in 50% (w/v) sucrose in PM buffer [50 mM HEPES (pH 7.0), 140 mM NaCl, and 3 mM MgCl_2_], sonicated, and centrifuged. cTMT-L-expressing membranes in the supernatants were precipitated by a three-fold dilution with PM buffer. A G protein activation assay was carried out as described elsewhere [16]. Go-type G protein was purified from pig cerebral cortex according to Katada et al.’s method [17]. The cTMT-L-expressing membranes were mixed with G protein solutions and then pre-incubated for 1 h at 0°C. Samples were irradiated with light from a 1-kW tungsten-halogen projector lamp that had passed through the following combined interference/cut-off filters: BP400 (Kenko Tokina Co., Ltd.; Tokyo, Japan; http://www.kenkoglobal.com/) + L39 (Toshiba; Tokyo, Japan); KL42 + L39 (both Toshiba), BP440 (Kenko) + L39 (Toshiba); BP450 (Kenko) + L39 (Toshiba); BP460 (Kenko) + Y44 (Toshiba); BP470 (Kenko) + Y44 (Toshiba); BP480 (Kenko) + Y44 (Toshiba); BP500 (Kenko) + Y44 (Toshiba); KL52 + Y44 (both Toshiba); BP540 (Kenko) + Y44 (Toshiba); and KL58 + Y44 (both Toshiba). The photon density at the sample location in each light condition was 2.3 × 10^13^ photons/mm^2^, as measured by an optical power meter (Nova laser power meter and energy meter; Ophir Optronics; Jerusalem, Israel) with a power sensor (30A-P-17; Ophir Optronics). After irradiation, the GDP/GTPγS exchange reaction was started by adding GTPγS solution to the pigment and G protein mixture. The final assay mixture consisted of 50 mM HEPES (pH 7.0), 140 mM NaCl, 1 mM MgCl_2_, 5 mM DTT, 250 nM GTPγS, 5 μM GDP, and 500 nM G proteins. The reaction was terminated after 1 min by adding stop solution (20 mM Tris/Cl, pH 7.4, 100 mM NaCl, 25 mM MgCl_2_, 1 μM GTPγS, and 2 μM GDP) and immediate filtering of the sample through a nitrocellulose membrane to trap G protein-bound [^35^S]GTPγS. Membrane-associated [^35^S]GTPγS was quantitated by a liquid scintillation counter (Tri-Carb 2910 TR; PerkinElmer; Waltham, MA).

### Fixation and sectioning

After being perfused with ice-cold 4% paraformaldehyde in phosphate-buffered saline (PBS), chicken heads were dissected quickly and the anterior segment of each eye was removed. The dissected heads were postfixed for 3 h at 4°C and then transferred to 20% sucrose until they sank. The tissue samples were embedded in optimal cutting temperature compound (Sakura Finetek Japan Co., Ltd.; Tokyo, Japan) and then cut into 20-μm sections with a cryostat (Leica Biosystems; Wetzlar, Germany). The sections were thaw-mounted onto SuperFrost Plus slides (Fisher Scientific; Pittsburgh, PA), dried at room temperature, and stored at -30°C until they were analyzed.

### *In situ* hybridization (ISH)

RNA probes for *cOpn3* and *cTMT* were generated from partial 761-bp and 656-bp cDNA fragment templates, respectively. Digoxigenin-labeled antisense and sense control riboprobes were generated by *in vitro* transcription. ISH was carried out on 20-μm-thick frozen sections according to published protocols [18]. For double staining with anti-Lhx1 antibody, retinal sections not subjected to proteinase K digestion during ISH were labeled with primary antibody (anti-Lhx1; S4 Table), followed by Alexa Fluor 488-conjugated anti-mouse secondary antibody diluted 1:750 in PBS. Micrographs were taken with a Nikon digital camera (DS-Ri1; Nikon; Tokyo, Japan) mounted on a Leica microscope (DM5000B) and processed in Adobe Photoshop CS 5.1 (Adobe Systems Inc., San Jose, CA).

### Antibodies

Specific polyclonal antibodies were raised in guinea pigs to the C-termini of cOpn3, cTMT-S, and cTMT-L. Each antibody was raised against a C-terminal 17-amino-acid synthetic peptide conjugated to keyhole limpet hemocyanin by Tanpaku-Seisei-Kogyo Co., Ltd. (Gunma, Japan; http://pro-purify.co.jp/laboratory/), according to their standard procedures. The resultant antibodies were affinity purified with antigen peptides by Tanpaku-Seisei-Kogyo. We confirmed that the pre-immune sera gave no signals similar those of post-immune sera. The characteristics of the primary and secondary antibodies used are summarized in S4 Table. We evaluated the specificity of the primary antibodies by comparison with published results. Sections incubated with secondary antibodies alone were devoid of fluorescence, indicating that signals observed in sections incubated with primary antibodies were not due to nonspecific binding of secondary antibody or tissue autofluorescence.

### Western blotting

Chicken embryonic tissues (heart, retina, and cerebellum at E19) were dissected and snap-frozen in liquid nitrogen. Frozen tissues were ground up with a pestle in a chilled mortar and solubilized immediately in RIPA buffer [10 mM Tris-HCl (pH 7.4), 1% octylphenoxypolyethoxyethanol, 0.1% deoxycholate, 0.1% sodium dodecyl sulfate (SDS), 0.15 M NaCl, 1 mM ethylenediaminetetraacetic acid]. Solubilized tissues were sonicated and centrifuged at 19000 ×*g* at 4°C for 15 min. The tissue lysate was subjected to SDS-polyacrylamide gel electrophoresis (PAGE), transferred onto a polyvinylidene difluoride membrane, and probed with anti-cOpn3 or anti-cTMT-L antibody, both diluted 1:3,000 in Immuno Shot (Cosmo Bio Co. Ltd.; Tokyo, Japan). After washing, secondary antibody (anti-guinea pig IgG) was added at 1:10,000 dilution and signals were detected by with an ImmunoStar LD (Wako Pure Chemical Industries Ltd.; Osaka, Japan). To ascertain immunolabeling specificity, immunogen peptide preabsorption tests were performed with a solution in which the molar ratio of anti-cOpn3 or anti-TMT antibody and the indicated antigen peptides was 1:20.

### Immunohistochemistry (IHC)

Fluorescent immunolabeling was performed using standard techniques. Briefly, all slides were blocked for 30 min at room temperature in PBS Triton X-100 (0.25%) (PBST) with 5% goat serum (Vector Laboratories; Cambridgeshire, UK). Primary antibodies were diluted (1:500 for anti-cOpn3, 1:2000 for anti-cTMT-L) in PBST with 5% serum and secondary antibodies in PBS. All wash steps included three 5-min washes with PBST. Primary antibodies were incubated for 6 h at room temperature or for 16 h at 4°C. Secondary antibodies were incubated for 1.5 h at room temperature. For double fluorescent labeling experiments, the slides were incubated with primary antibodies (anti-cOpn3 or anti-cTMT-L) and secondary antibodies in a sequential manner: followed by anti-Lhx1 or other antibodies. The slides were mounted with anti-fade mountant supplemented with 4', 6-diamidino-2-phenylindole (DAPI) (Vector Laboratories). We collected fluorescent images using a Leica TCS-SP5 or a Zeiss LSM 780 (Carl Zeiss Microscopy GmbH; Jena, Germany) confocal laser-scanning microscope with 405-nm, 488-nm, and 543-nm excitation wavelengths and 424–489-nm, 505–539-nm, and 551–618-nm emission wavelengths for DAPI, green, and Cy3, respectively.

## Results

### Comparison of the genomic structures of opsin 3-related genes

**Fig 1:**
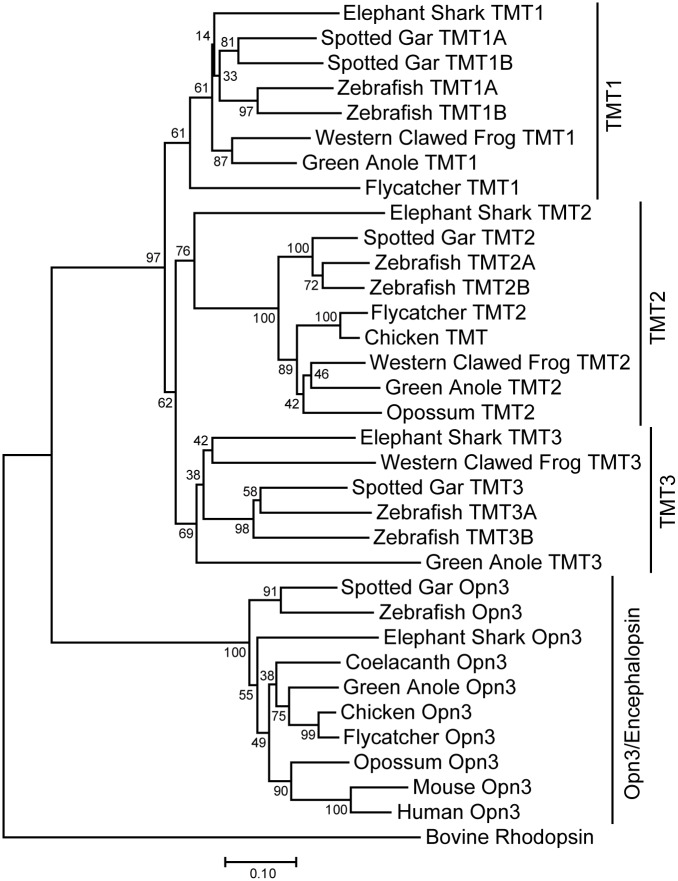
Phylogenetic tree of opsin 3-related proteins. The opsin proteins are depicted by the neighbor-joining method. Bovine rhodopsin was used as the outgroup. The scale bar is calibrated to substitutions per site. Numbers show bootstrap confidence values. Amino acid sequences used in the tree construction were deduced from the nucleotide sequences listed in S2 Table.

**Fig 2:**
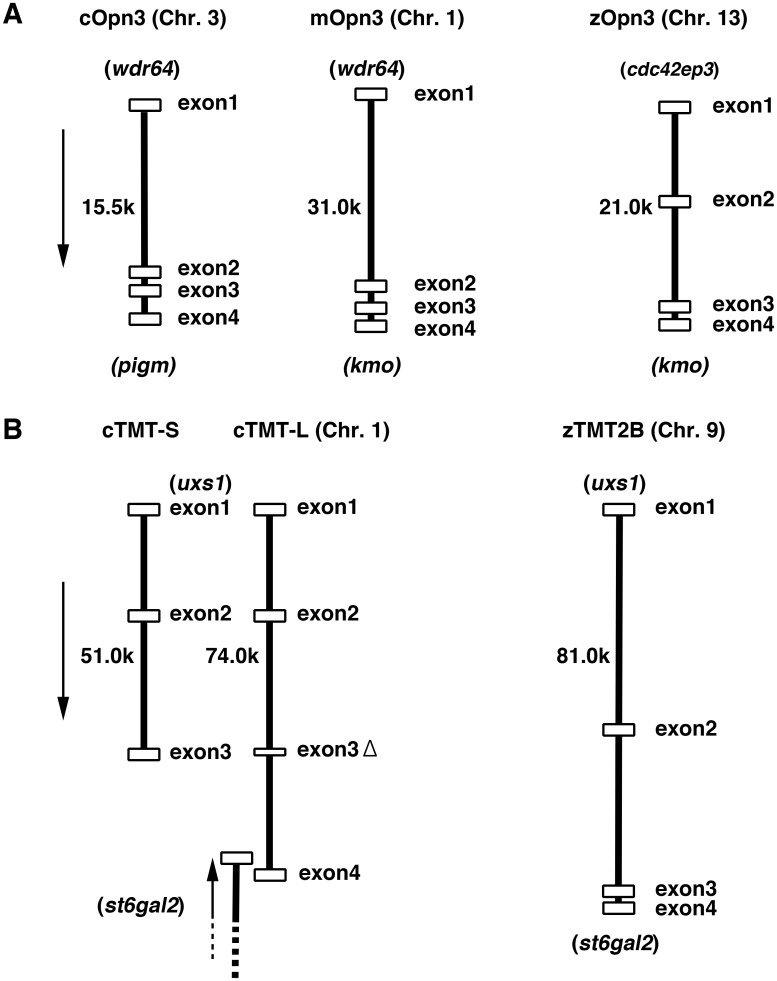
Intron-exon structure of *Opn3* (A) and *TMT* (B) in chicken, mouse, and zebrafish. Exons and introns are indicated with open boxes and solid bars, respectively. Approximate gene sizes are shown. Flanking genes (see S3 Table) are shown in parenthesis. Direction of transcription is indicated with an arrow. Note that the fourth exon of cTMT-L is in the intron of the next gene, *st6gal2*.

Based on phylogenetic comparisons, we determined that two novel *opsin 3*-related genes found in the chicken genome should be classified as an Encephalopsin (Opn3) and TMT2, respectively (Fig 1). Here we call them *cOpn3* and *cTMT*, respectively. Comparisons of the intron-exon structures of *cOpn3* and *cTMT* with mouse and zebrafish genes recorded in genomic databases (Fig 2) revealed that *cOpn3* consisted of four coding exons with an intron-exon structure consistent with that in mouse and zebrafish homologues (Fig 2A). Meanwhile, we found that *cTMT-S* and *cTMT-L*, from the same gene, consisted of three and four exons, respectively (Fig 2B). A phylogenetic tree of opsin 3-related proteins from 11 species showed that there were at least three TMT opsin subgroups, TMT1, TMT2, and TMT3 as reported previously (Fig 1, S2 Table) [9, 19]. The novel chicken TMT opsin belongs to the TMT2 subgroup (Fig 1A). Comparison of the flanking genes of *cTMT* with those of a zebrafish *TMT2* gene, *zTMT2B* (JX293362), showed that *cTMT* shares synteny with *zTMT2B* (Fig 2B). The intron-exon junctions in *cTMT-L* at nucleotides 337, 662, and 895 were found to be similar to those of *zTMT2B* at nucleotides 366, 694, and 927, indicating that intron-exon structure was conserved between *cTMT-L* and *zTMT2B*.

**Fig 3:**
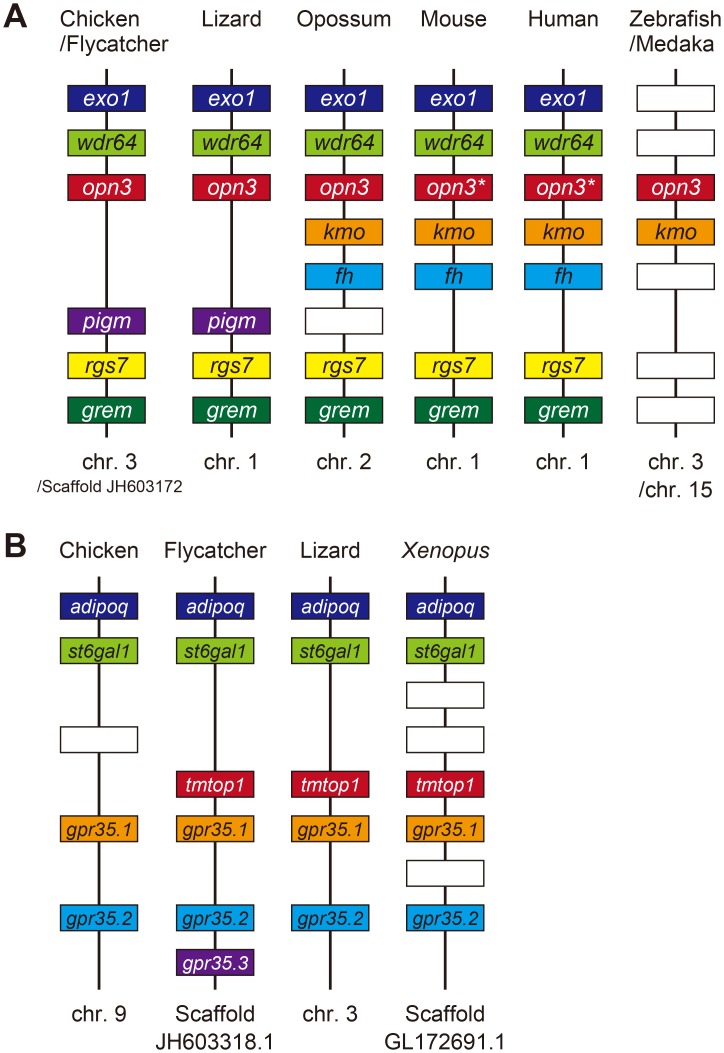
Syntenic regions encompassing the *Opn3* (A) and *TMT1* (B) loci. The *Opn3* and *TMT1* genes are indicated as *opn3* and *tmtop1* in red, respectively. The same colored boxes designate orthologous genes. Open boxes indicate non-orthologous gene displacement. Gene products and symbol definitions are listed in S3 Table.

We further compared the genes flanking *cOpn3* with those flanking *opn3* genes of different species indexed in the Ensembl database (Fig 3A, S3 Table). The *kmo* and *fh* genes are located between *opn3* and *rgs7* in the genomes of marsupials (opossum) and eutherians (mouse and human), but the loci of *wdr64* and *rgs7* are conserved between sauropsid (birds and reptiles) and human genomes. In zebrafish, most of the flanking genes are different, except for *kmo*. This gene order surrounding the *zOpn3* gene was conserved in the medaka genome. By contrast, the genes flanking c*TMT*, namely *uxs1* and *st6gal2*, were found to be conserved relative to zebrafish (*zTMT2B*) (Fig 2B) and clawed frog TMT2 (data not shown). Two types of TMT opsin genes, TMT1 and TMT2, have been observed in birds [20]. In the flycatcher and lizard genomes, the *tmtop1 (TMT1)* locus is situated between the *st6gal1* and *gpr35* loci (Fig 3B). Our Blast search indicated that an uncharacterized 131-amino-acid protein, similar to DNA-(apurinic or apyrimidinic site) lyase 2, is encoded at this locus in the chicken genome. Altogether these comparisons indicate that *Gallus gallus* has two opsin 3-related genes, *Opn3* and *TMT2* orthologs belonging to the opsin 3 group.

### Key features of opsin 3-related proteins

Multiple alignment of the amino acid sequences of opsin 3-related proteins revealed several conserved key features (S1 Fig). These features include seven putative transmembrane α-helices and a lysine residue at position 296 (in the bovine rhodopsin numbering system) required to form the Schiff base with the chromophore, which is characteristic of opsin proteins. The canonical motifs of rhodopsin-like G protein-coupled receptors, such as the (D/E)R(Y/W) motif at the end of transmembrane domain 3 (TM3), the CWxP motif in TM6, and the NPxxY motif in TM7, are conserved (S1 Fig). The positively charged Schiff base is balanced by a counterion from an acidic amino acid, which was postulated to be an aspartate residue at position 113 (D113) in TM3 [21, 22], or a glutamate or aspartate residue at position 181 (E/D181) in the second extracellular loop [23] in Opn3. By contrast, an aromatic amino acid tyrosine occupies position 113 (Y113) in the TMT group. Thus a conserved E181 can function as the counterion in TMT opsins.

### Photosensitivity of cTMT protein

**Fig 4:**
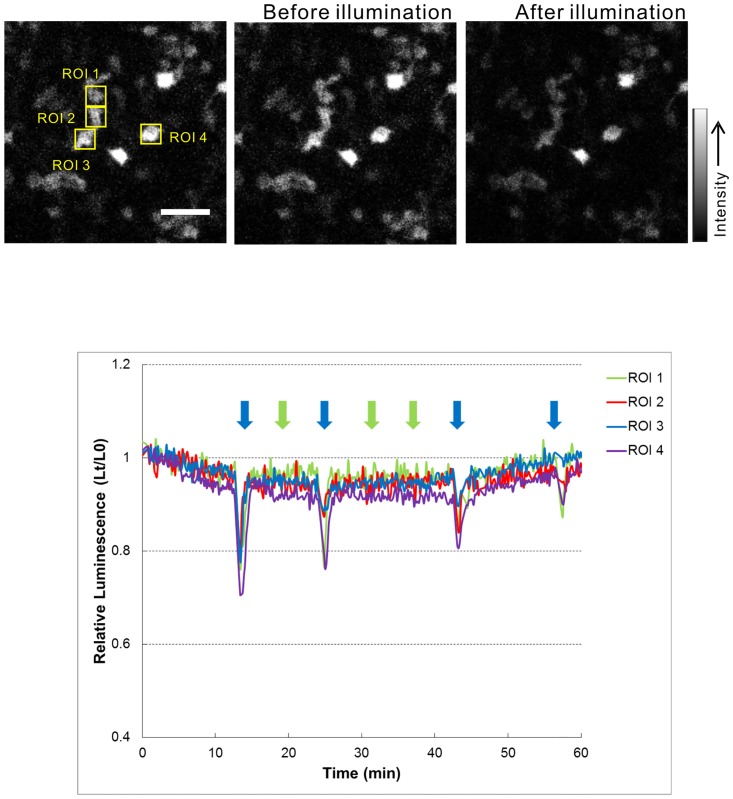
Ca^2+^ responses after light stimulation of *cTMT-L*-expressing cells. Cells transfected with both cTMT-L and cpGL-CaM were treated with 11-cis-retinal (5 μM) and luciferin (2 mM). Luminescence intensity in images acquired every 10 s was calculated relative to that measured at imaging onset (Lt/L0) for cells in the indicated ROI (upper left) as shown in the trace. Representative bioluminescence images acquired before (upper center) and after (upper right) illumination are shown. Relative bioluminescence signals (Lt/L0) of cTMT-L-expressing cells decreased following blue light pulses (470–490 nm; 10 s at 0.2 μW/mm^2^; blue arrows), but not with green light pulses (535–555 nm; 10 s at 0.2 μW/mm^2^; green arrows). Representative imaging data from three independent experiments are shown. Scale bars: 50 μm.

Luminescence was visualized in Neuro2a cells transfected with cpGL-CaM and cTMT-L, cTMT-S, or cOpn3 expression vectors. Upon 10-s stimulation with blue light through a band-pass filter (470–490 nm, 0.2 μW/mm^2^), a transient decrease in luminescence was observed in Neuro2a cells containing 11-cis-retinal-reconstituted cTMT-L (Fig 4). However, upon stimulation with green light through a band-pass filter (535–555 nm), the cells did not exhibit light-induced changes in the bioluminescence of the indicator (Fig 4). On the other hand, no bioluminescence changes were observed in cells transfected with cTMT-S or cOpn3 in either condition (not shown). These results suggest that retinal-reconstituted cTMT-L serves as a blue-light absorbing photoreceptor involving Ca^2+^ signaling.

**Fig 5:**
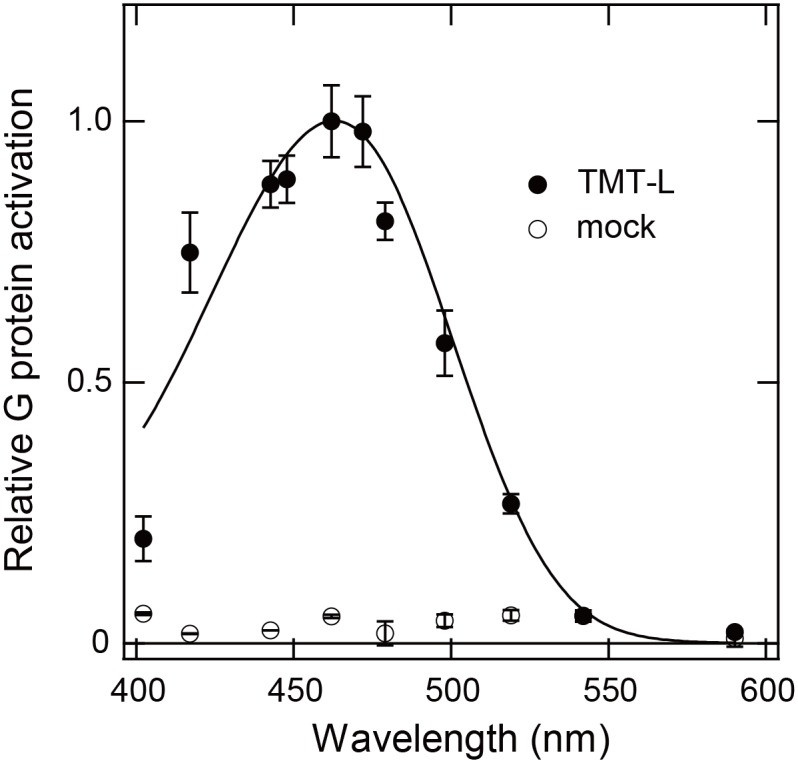
Wavelength dependence of Go activation efficiencies of cTMT-L. An action spectrum was constructed from wavelength-dependent Go activation efficiencies of cTMT-L-transfected (closed circle) or mock-transfected (open circle) HEK293T cell membranes. G protein activation efficiency was measured by irradiation with eleven different light wavelengths of various intensities (S2 Fig). The relative Go activation efficiencies obtained by irradiation at around 2.3 × 10^13^ photons/mm^2^ were calculated by normalizing the maximum value to 1.0. The data were best fitted to a Govardovskii template with a λmax of 463 nm (solid curve). All data represent the mean ± S.D. of four independent experiments.

We found that membranes expressing recombinant cTMT-L proteins reconstituted with 11-*cis* retinal activated Go-type G protein in a light quantity-dependent manner in HEK293T cells (S2B Fig). Blue light (~470 nm) was particularly effective for G protein coupling by cTMT-L (S2A Fig). Based on G protein activation levels induced by various wavelengths of light, we plotted relative G protein activation efficiency as a function of peak wavelength of light transmitted through band-pass filters. The action spectrum for G protein activation fitted with a Govardovskii template [24] peaked at 463 nm (Fig 5, solid curve), a wavelength similar to the absorption spectra of fish TMT opsins [16, 19].

### Localization of *cTMT* mRNA in the embryonic and hatchling retina

Because we were able to isolate partial *cOpn3* and *cTMT* cDNA fragments from E17 retina, a few days before hatching, we first examined the expression pattern of both mRNAs in E17 retina. ISH performed with an RNA probe generated from a *cTMT* cDNA fragment common to both cTMT-S and cTMT-L, and thus unable to discriminate between the two, revealed that *cTMT* mRNA was expressed by subsets of cells in the inner nuclear layer where retinal interneurons reside (S3A Fig). In contrast, *cOpn3* mRNA was not detected by ISH in the E17 retina (S3C Fig).

**Fig 6:**
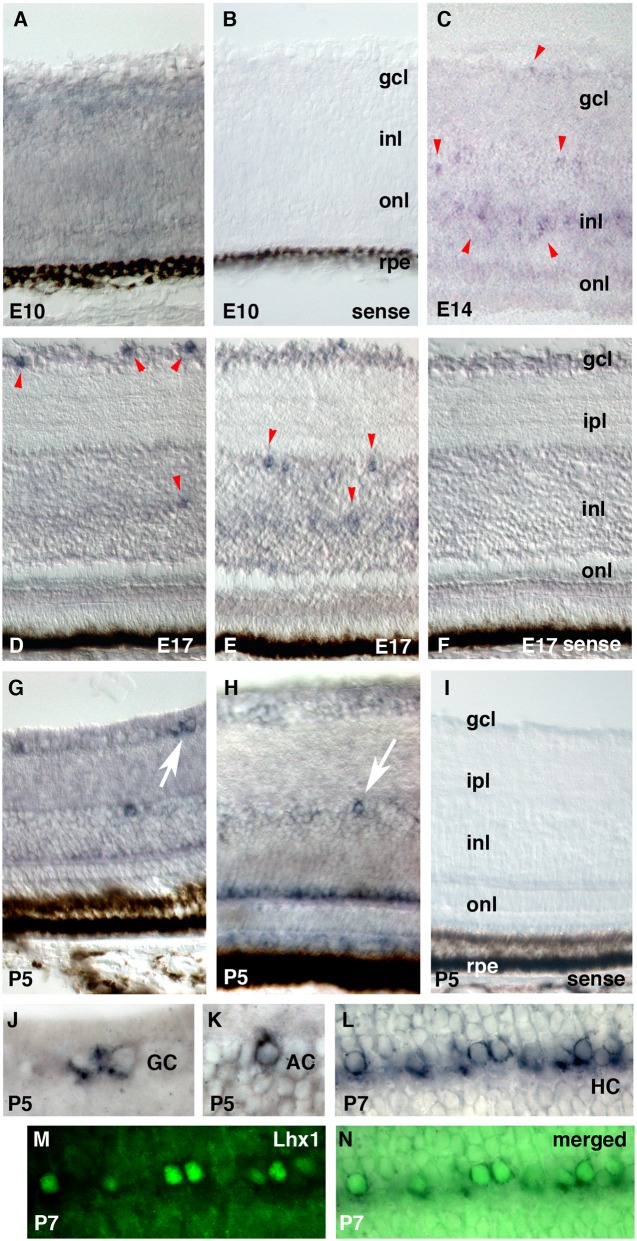
*In situ* hybridization of *cTMT* in the developing and post-hatch retina. Central retina is shown, vitreal side up, unless otherwise indicated. Negative sense-probe controls are shown in panels **B, F**, and **I**. **(A)** Diffuse *cTMT* mRNA in the E10 retina. **(C)** *cTMT* expression in subsets of cells in the gcl and inl at E14. **(D, E)** *cTMT*-expressing cells (arrowheads) in the gcl or inl are distinctly observed at E17. **(G, H)** *cTMT* expression in HCs at P10. Putative ganglion and amacrine cells (arrows) are enlarged in panels **J** and **K**, respectively, and peripheral retina is shown in panel **G**. **(L-N)** The same sections, showing the HCs with *cTMT* mRNA shown in blue **(L)**, Lhx1 protein shown in green **(M)**, and a merged view **(N)**. Abbreviations: gcl, ganglion cell layer; inl, inner nuclear layer; ipl, inner plexiform layer; onl, outer nuclear layer; rpe, retinal pigment epithelium; GC, putative ganglion cell; AC, putative amacrine cell; HC, putative horizontal cell.

Because non-canonical *opsin* mRNA has been detected earlier in development than classical photopigments [25], we performed ISH on a series of developing retinal sections to detect the timing of the developmental onset of *cTMT* expression. At E7, *cTMT* mRNA was not detected in the retina (not shown). By E10, *cTMT* was expressed diffusely (Fig 6A); the negative control experiment with a sense probe did not produce any signal (Fig 6B). At E14, *cTMT* was expressed by subsets of cells in the ganglion and inner nuclear layers (Fig 6C). At E17, *cTMT* mRNA was detected clearly in small subsets of cells in the ganglion cell layer (Fig 6D), and the innermost and middle regions of the inner nuclear layer (Fig 6E and 6F for a negative control). By day 5 post-hatching (P5), *cTMT* was expressed in the outermost layer of the inner nuclear layer, where horizontal cells (HCs) reside, as well as by a small subset of cells in the ganglion cell and inner nuclear layers (Fig 6G–6I, 6J and 6K). Examination of the sections under high magnification revealed that not all HCs were positive for *cTMT* mRNA (Fig 6L).

To identify whether *cTMT*-expressing retinal HCs in chickens are the axon-bearing or axon-less HC morphology type [26], we performed IHC with an antibody targeting Lhx1, a homeobox gene-encoded protein specific to axon-bearing HCs [27], following *in situ* hybridization with our *cTMT* probe. We found that *cTMT*-expressing HCs were Lhx1-IR (Fig 6L–6N), demonstrating *cTMT* expression by axon-bearing HCs in the post-hatch chicken retina.

### Localization of cTMT protein in the retina

Opsin 3 protein localization in the chicken retina was examined with polyclonal anti-cOpn3 and anti-cTMT antibodies raised by our group. Because it was reported recently that different TMT isoforms might exhibit distinct distribution in the retina [28–30], we attempted to raise separate cTMT-S- and cTMT-L-specific antibodies, but did not succeed in raising specific anti-cTMT-S antibodies. Our antibodies were validated in western blot analyses (S4 Fig). Bands at the predicted molecular weights of cTMT-L (~41 kDa) and cOpn3 (~43 kDa) were detected in the retina and cerebellum just before hatching (S4A and S4B Fig). Blots incubated with antigen-absorbed antibodies yielded no bands at corresponding molecular weights (S4A and S4B Fig).

**Fig 7:**
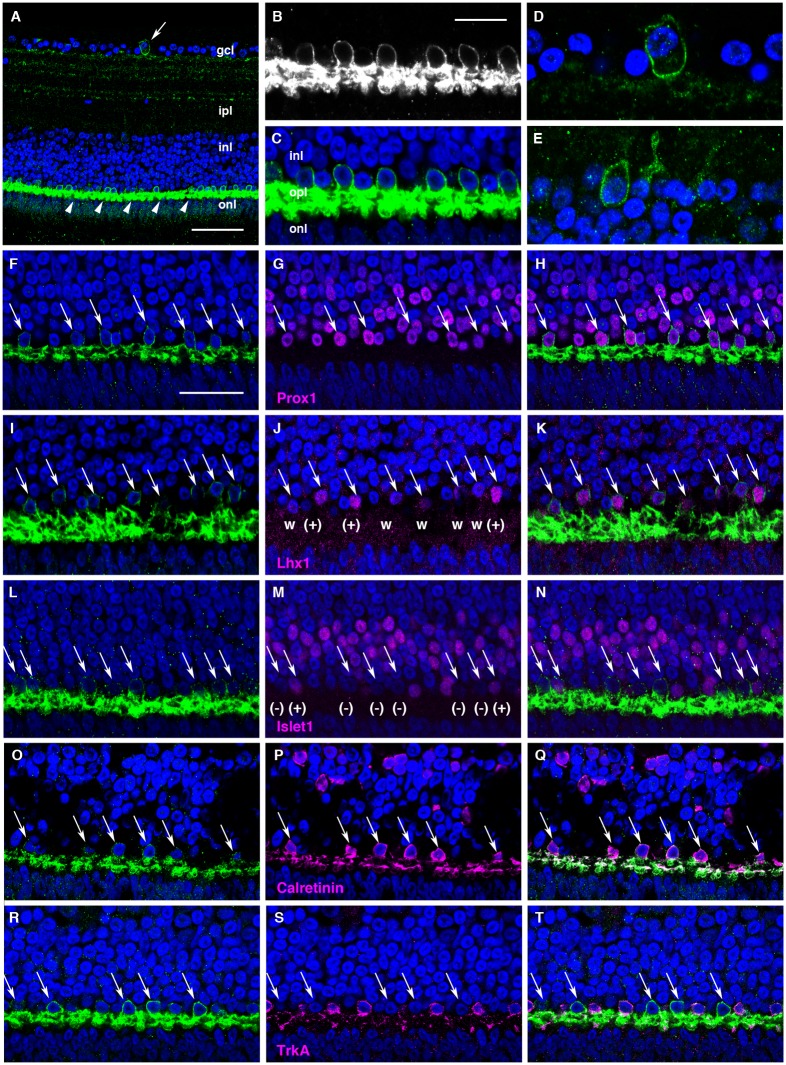
Localization of cTMT-L protein in P10 retina. Immunoreactivity for cTMT-L is shown in green (white in panel **B**), and DAPI counterstained nuclei appear blue. **(A)** A cTMT-IR cell in the gcl (arrow). Intense cTMT-L labeling in the processes of HCs in the opl (arrowheads). **(B)** Highly magnified cTMT-IR HCs. Note the intense signal in the processes of opl HCs. **(C)** Merged image of section highlighted in **B** with DAPI nuclear counterstain. **(D)** Highly magnified cTMT-IR cell in the gcl (arrow in **A**). **(E)** cTMT-IR cells in the inl abutting the ipl. **(F-T)** Sections double labeled for cTMT-L (left panels) and HC markers (middle panels). Merged images are shown in the right panels. Arrows highlight cTMT-IR HCs. In **J**, there are Lhx1-positive (+) and weakly positive (w) cells among cTMT-IR cells. In **M**, there is a mixture of Islet1-positive (+) and -negative (-) cTMT-IR cells. Abbreviations: gcl, ganglion cell layer; inl, inner nuclear layer; ipl, inner plexiform layer; onl, outer nuclear layer; opl, outer plexiform layer. Scale bars: 50 μm in **A**, 12.5 μm in **B-E**, and 25 μm in **F-T**.

IHC of the retina showed the distinct presence of cTMT-L in P10 HCs (Figs 7A–7C and S5C); no signal was detected in the negative control experiment when antigen-absorbed anti-cTMT-L antibody was used (S5D Fig). cTMT-L- immunoreactivity was observed in somata aligned at the outermost layer of the inner nuclear layer, and in their brush-shaped processes (Fig 7B and 7C). Relatively few cTMT-immunoreactive (IR) cells were found in the ganglion cell layer (Fig 7A and 7D, high magnification) and inner nuclear layer (Fig 7E).

### Clarification of cTMT-positive HCs in the retina

To investigate the type of cTMT-IR HCs in the P10 retina, we performed double-IHC with antibodies raised against the HC markers Islet1, Calretinin, and TrkA together with anti-Lhx1 antibodies (Figs 7F–7T and S6). cTMT-IR cells were first labeled with the pan-HC antibody anti-Prox1 [27]. We confirmed that all cTMT-L-IR HCs were Prox1-positive, though there were cTMT-L-negative, Prox1-positive HCs (Fig 7F–7H). We observed a weak Lhx1-labeling subpopulation among the cTMT-IR HCs (Fig 7I–7K). With a few exceptions, most cTMT-IR HCs were Islet1-negative (Fig 7L–7N).

IHC for expression of Calretinin, characteristic of brush-shaped axon-bearing HCs, and anti-TrkA, characteristic of axon-less HCs, [27, 31] indicated that cTMT-IR HCs were Calretinin-positive (Fig 7O–7Q) and TrkA-negative (Fig 7R–7T). Furthermore, the cTMT-IR HCs had a brush-shaped morphology (Fig 7O) consistent with that found in Calretinin-positive HCs (Fig 7P). Because most Calretinin-positive HCs are Lhx1-positive (~52% of all HCs), with a small number being Islet1-positive (~8%) [27], these findings indicate that cTMT-L was localized in Calretinin-positive, brush-shaped axon-bearing HCs.

### Localization of cOpn3 protein in the retina

**Fig 8:**
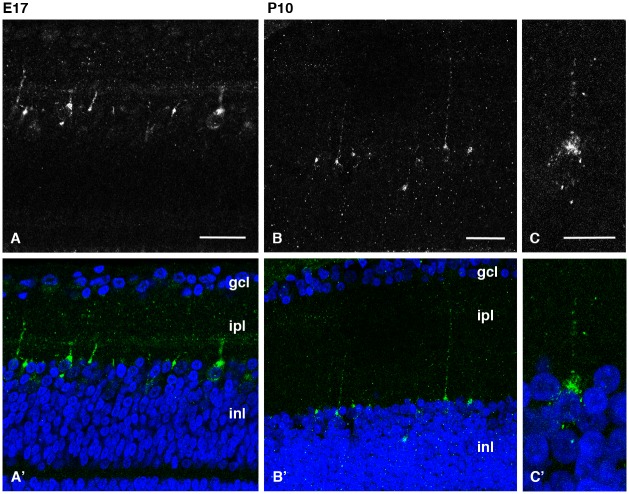
Localization of cOpn3 protein in E17 (A, A’) and P10 (B-C’) retina. cOpn3 immunoreactivity is shown in white in the upper panels **(A–C)** and in green together with DAPI nuclear counterstain in blue in the lower panels **(A’-C’)**. Highly magnified cOpn3-IR cell are shown in **C/C’**. cOpn3-IR cells can be seen in the inl abutting the opl with processes directed upward toward the ipl. Typically, each cOpn3-IR cell is heavily labeled in the soma from which a single long process emerges. Z-stack images (14 μm for **B/B’**, 13 μm for **D/D’**). Abbreviations: gcl, ganglion cell layer; ipl, inner plexiform layer; inl, inner nuclear layer. Scale bars: 100 μm in **A**, 25 μm in **B** and **C**, and 10 μm in **D**.

Although *cOpn3* mRNA was not detected in the retina, IHC revealed cOpn3 protein expression in a subset of inner nuclear layer cells at E17 (Figs 8A/8A’ and S5A) and P10 (Fig 8B–8C’). Typically, cOpn3-IR retinal cells are heavily labeled in the soma from which a single long process emerges. This staining pattern was never observed when antigen peptide-absorbed anti-cOpn3 antibody was used in IHC (S5B Fig). The failure of our standard ISH experiment to detect *cOpn3* mRNA in the E17 retina (S3C Fig) although RT-PCR analysis showed expression of *cOpn3*, suggests that a very small amount of *cOpn3* mRNA is expressed in the retina whereby accumulation of the protein enabled detection of cOpn3-IR cells. Alternatively, this anti-Opn3 antibody is polyclonal and might cross-react to other proteins than Opn3 as revealed by multiple bands in Western blot analysis (S4B Fig).

### Localization of cOpn3 and cTMT proteins in the brain

**Fig 9:**
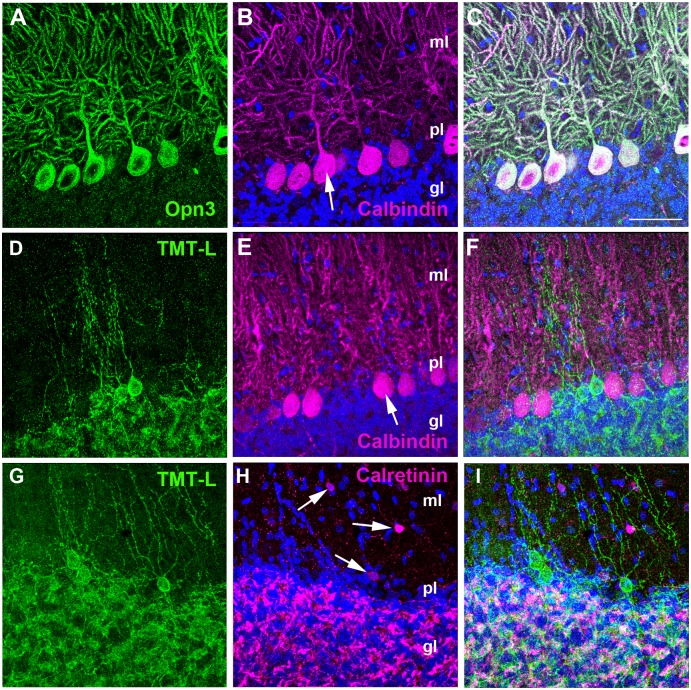
Localization of cOpn3 (A-C) and cTMT (D-I) proteins in P10 cerebellum (sagittal sections). cOpn3 or cTMT, Calbindin (Purkinje cell marker) or Calretinin (stellate and basket cell marker), and cell nuclei (DAPI) are shown in green, magenta, and blue, respectively. **(A-C)** cOpn3 is localized to Calbindin-IR Purkinje cells. Arrow in **B** shows a soma of a Purkinje cell. **(D, G)** cTMT-L is present in small Calbindin-negative cells near Purkinje cells and in neurofibers in the cerebellar granule cell layer. **(E)** Calbindin-labeled Purkinje cells. Arrow in **E** shows a soma of a Purkinje cell. **(F)** Calbindin-negative cTMT-IR cells. **(H)** Calretinin-labeled stellate and basket cells (arrows) in the molecular layer. Mossy fibers and cerebellar glomeruli in the granule cell layer are also labeled by Calretinin (magenta). **(I)** Calretinin-negative cTMT-IR cells. Abbreviations: ml, molecular layer of the cerebellum; pl, Purkinje cell layer; gl, granule cell layer. Scale bar: 50 μm in all. Z-stack images (10 μm) are shown in (**D-F, G-I**).

We next examined whether cOpn3 and cTMT-L proteins were present in the chicken brain given that previous studies revealed encephalopsin mRNA in the mouse brain [6, 32] and TMT opsin in the zebrafish brain [9]. We found that cOpn3 protein was localized to Purkinje cells of the cerebellar cortex at P10 (Fig 9A–9C). We also found that cTMT-L protein was present close to the Purkinje cell layer and in neurites located in the granule cell layer (Fig 9D–9F). In the vicinity of Purkinje cells, there are basket cells in the molecular layer and Golgi cells in the granule cell layer [33]. Double staining showed that cTMT-IR cells were negative for the stellate/basket cell marker [34, 35] Calretinin (Fig 9G–9I). Thus, we deduced that the cTMT-IR cells in the chicken cortex were likely Golgi cells in the granule cell layer. cTMT-immunoreactivity was also observed in mossy fibers and cerebellar glomeruli (Fig 9G), with partial colocalization with Calretinin (Fig 9H and 9I) [35].

**Fig 10:**
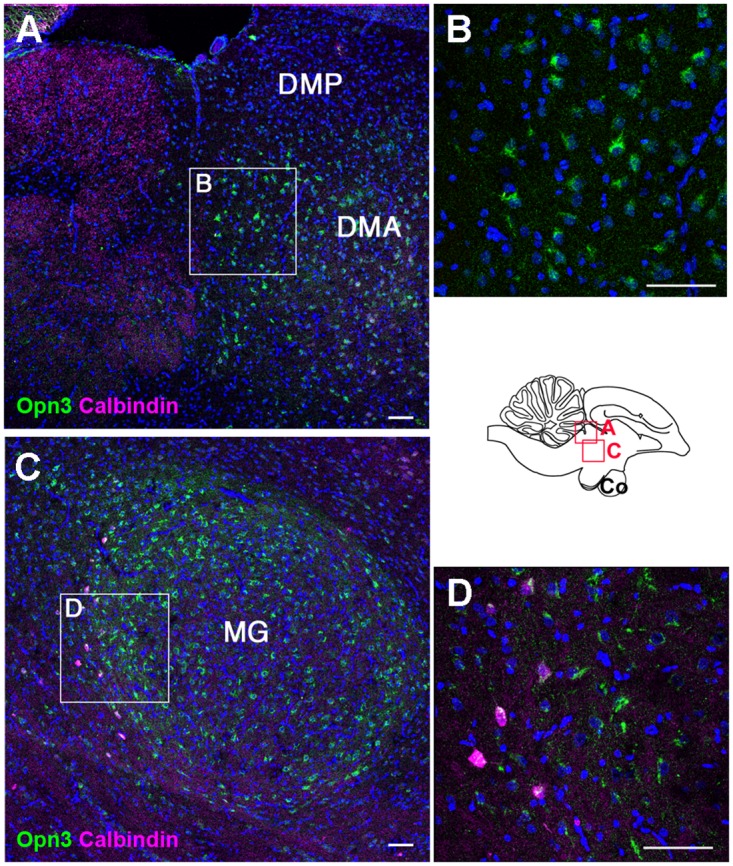
Localization of cOpn3 protein in P10 thalamus (sagittal sections). Expression of cOpn3 in the dorsomedial anterior nucleus (DMA) **(A)** and medial geniculate nucleus (MG) **(C)** of the thalamus. High magnification views of boxed areas in **A** and **C** are shown in **B** and **D**, respectively. Calbindin-IR neurons are present posterior to the MG (**C**). Z-stack images (7 μm for **B**, 4 μm for **D**). Abbreviations: DMA, dorsomedial anterior nuclei; DMP, dorsomedial posterior nuclei; MG, medial geniculate nuclei. Scale bars: 50μm.

**Fig 11:**
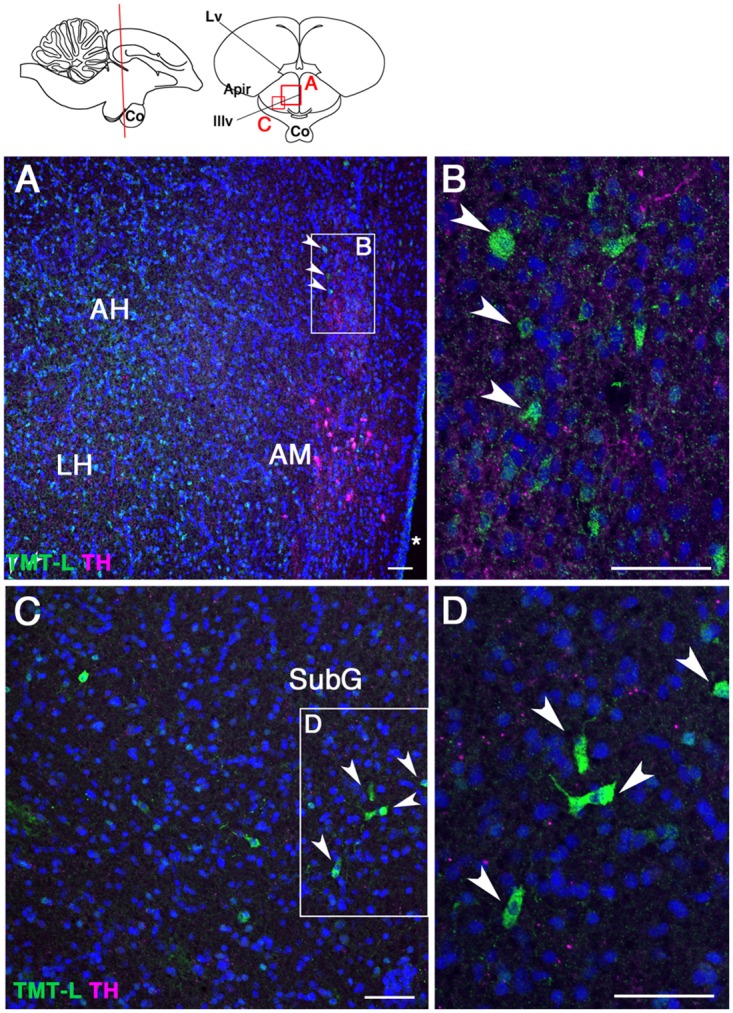
Localization of cTMT protein in P10 anterior hypothalamus (coronal sections). **(A)** Arrowheads show cTMT-IR neurons dorsal to tyrosine hydroxylase (TH)-IR neurons and lateral to the third ventricle (asterisk). **(B)** Highly magnified cTMT-L-IR neurons. **(C)** cTMT-IR neurons in the subgeniculate nucleus (SubG). **(D)** Highly magnified cTMT-IR neurons (arrowheads in **C**). Abbreviations: AH, anterior hypothalamic area; AM, nucleus anterior medialis hypothalami, Apir, amygdalo-piriform transition area; Co, optic chiasm; LH, lateral hypothalamic area; Lv, lateral ventricle; Pa, paraventricular nucleus; IIIv, third ventricle. Scale bar: 50 μm.

Encephalopsin mRNA has been detected in mouse thalamic nuclei and cerebellum [6]. We found that cOpn3 was expressed by a subset of cells in the dorsomedial (Fig 10A and 10B) and medial geniculate (Fig 10C and 10D) nuclei of the thalamus. cTMT-L immunoreactivity was not observed in any thalamic nuclei (data not shown), but was found in subsets of cells in the paraventricular nucleus of the hypothalamus, dorsal to tyrosine hydroxylase-IR neurons in the nucleus anterior medialis hypothalami (Fig 11A and 11B), and in the subgeniculate nucleus (Fig 11C and 11D). cTMT-L IR was not detected in the light-sensitive paraventricular organ (S7 Fig).

## Discussion

Our phylogenetic tree of opsin 3-related proteins indicated that most vertebrate species have one encephalopsin (Opn3)-like protein, whereas the number of TMT opsin-like proteins varies across species (Fig 1 and S2 Table) [9,16,19]. In clawed frogs, *Xenopus tropicalis*, the *Opn3*-like gene (XM_002935666) possesses a lysine-to-isoleucine substitution at a site essential to retinal binding, implying that it has become a pseudogene. There are no TMT opsin-like proteins in eutherians, one TMT opsin-like protein in marsupials, one or two in birds, and three in lizard, frog, and shark. In teleosts, three TMT opsin paralogs are further diversified [16]. Our phylogenetic tree is largely consistent with the findings of recent reports [9,16,19]. Alignment of opsin 3-related proteins shows that the amino acid sequence for the C-terminal region of TMT opsins is diversified across species; and the two TMT isoforms in chicken indicates further diversification within the same animals. By contrast, the amino acid sequence of Opn3 is well conserved (S1 Fig). Thus, the molecular properties of Opn3 proteins may be conserved, while those of TMT opsins may vary across species, perhaps in relation to animals’ light environments or habitats.

Our genomic analysis confirmed that chicken TMT opsin belongs to the TMT2 subgroup (Figs 1 and 2B). Since marsupials have retained TMT2, while the eutherians have no *TMT* genes, TMT2 opsin might confer some advantage in the species in which it is retained. The loss of the *TMT* genes in eutherian mammals may be related to the evolution of Placentalia reproduction mode.

In spite of its encoding protein structures being conserved among sauropsids, marsupials, and mammals, *Opn3* has undergone some rearrangements on the flanking side where the *pigm/kmo* gene resides (Fig 3A). Given the *opn3* to *kmo* juxtaposition in fish genomes, it is conceivable that *opn3* and *kmo* first came to occupy loci between *wdr64* and *rgs7* during the evolution toward terrestrials. It appears, that *kmo* was then lost and, subsequently, *pigm* (in sauropsids) or *fh* (in marsupials and eutherians) was acquired. These genomic rearrangements may affect the species-specific expression of *Opn3*. Human *Opn3* gene has been observed to be widely expressed across tissues, including the retina [7], while mouse *Opn3* is expressed exclusively in the brain and testis [6, 32]. Although synteny (conserved flanking gene order) cannot explain this distinct expression pattern in mammals, susceptibility to gene arrangement might correlate with diversification of cis-elements that drive the expression of *Opn3*.

The discovery of intrinsically photosensitive retinal ganglion cells has overthrown the long-held belief that rods and cones are the exclusive retinal photoreceptors [2,36,37]. The present study shows that cTMT-L (a TMT2 opsin) is present in axon-bearing retinal HCs. Retinal HCs are interneurons that provide pathways for interactions between photoreceptors with adjustments in HCs themselves and from adjacent bipolar cells [26]. They have been known to have hyperpolarizing slow responses to light called S-potentials. In the pigeon retina, axon-bearing HCs connect to rods and cones via their axons and have inputs from cones on their dendrites [38]. Thus, it is conceivable that, in the chicken retina, axon-bearing HCs may respond directly to blue light via TMT opsin and modulate visual information processed by photoreceptors. A fish TMT2 opsin has been known to possess a unique molecular property: Its active state cannot photo-convert back to the resting state, which suggests that it can accumulate the active state in proportion to light intensity under weak light conditions and can completely convert to the active state under bright light conditions [16]. Thus, TMT2-expressing HCs may utilize such photoreceptive property for visual processing.

The chicken melanopsins Opn4m and Opn4x are also blue light sensors and Opn4x is localized to axon-less HCs [31,39–41]. Axon-less HCs in mammals and sauropsids have a preponderance of inputs from cones [26]. Furthermore, electrophysiological studies have shown that cone HCs are photosensitive and express melanopsin in teleost catfish [42]. Hence, retinal HCs can be regarded as non-classical photoreceptors that respond to blue light through TMT opsin or melanopsin photopigment in particular species.

A number of opsin-related genes are expressed in the developing retina [25, 39]. Here, we observed that opsin 3-related proteins are already present in the inner retina at E17 in chickens, as was found previously for a chicken opsin 5 gene [3]. It remains to be determined whether these non-canonical opsin-positive cells in the inner retina, especially cTMT-L-positive HCs, are functional photoreceptors before hatching or are present only in anticipation of hatching.

The avian hypothalamus, a deep brain structure, is photosensitive and exhibits expression of VA opsin and opsin 5 [43]. This study adds a third avian deep brain photoreceptor, namely a TMT opsin. The maximum absorption wavelengths for opsin 5 (chicken Opn5m), TMT opsin (Fugu), and VA opsin (frog) are 360 nm, 460 nm, and 501 nm, respectively [5,19,44]. Thus, the chicken brain may be responsive to ultra-violet, blue, and green light through photoreceptors in different hypothalamic nuclei. It will be intriguing to elucidate the functional differences between these suggested photoreception abilities. In particular, it will be important to determine whether these photoreception abilities have distinct physiological consequences.

Unfortunately, we were not able to determine with certainty whether the chicken Opn3, encephalopsin is photosensitive or not. Nevertheless, cOpn3 protein was localized to Purkinje cells of the cerebellum and thalamic nuclei in the chicken, similar to the expression observed in the mouse brain [6, 32]. It could be that chicken Opn3 protein mediates other roles, such as regulating apoptosis or immune modulation [45,46]. A recent study has shown that a chimeric chicken Opn3, in which the third intracellular loop was replaced with that of a jellyfish opsin, forms a blue-sensitive pigment [47]. Given that zebrafish Opn3 has a an absorption maximum at around 465 nm when it is expressed in mammalian cultured cells [47], the intact chicken Opn3 may retain the similar property.

Chicken melanopsin (cOpn4x) is expressed in the cerebellum [48]. Both cOpn4x [40] and cTMT are blue light sensors at least functioning in cultured cells. This study shows that cTMT is present in Golgi cells, and perhaps other cells, as well in the cerebellum. Since there have been no data indicating that the cerebellum is photoreceptive so far, it would be of great interest to elucidate photosensitivity of the chicken cerebellum *per se* and the functions of these blue light sensors *in situ*.

## Supporting Information

S1 Fig Alignment of predicted amino acid sequences encoded by *opsin 3*-related genes. Chicken TMT opsin (cTMT) and chicken encephalopsin (cOpn3) are aligned with other opsin 3-related proteins. Bovine rhodopsin is used as reference for counterion position. **(A)** Transmembrane domain 3 (TM3), intracellular loop 2 (ICL2), TM4, and extracellular loop 2 (ECL2) are shown. E113, (D/E)R(Y/W) motif, and E181 are highlighted. **(B)** TM6, ECL3, TM7, and C terminal domains are shown. CWxP motif, K296, and NPxxY motif are highlighted.
(PDF)

S2 Fig Wavelength dependence of Go activation efficiencies of chicken TMT. **(A)** Go activation efficiencies of cTMT-L in HEK293T cell membranes were measured by irradiation of selected wavelength light in the region of light intensity where linear relationships between Go activation efficiency and light intensity were mostly observed. The measurements were performed with lights of eleven different wavelengths at 0°C. The plotted data were calculated by subtracting the activity without light irradiation from that measured with irradiation within each light conditions. **(B)** Go activation profile of cTMT-L in HEK293T cell membranes following irradiation with 462 nm-light of different intensities. The data were fitted with a Hill equation: y = Basal + (Max − Basal) / (1 + EC_50_ / x), (Hill coefficient = 1, solid curve) and the EC_50_ value was calculated to be 2.12 × 10^13^ photons/mm^2^.
(TIF)

S3 Fig Expression pattern of chick *TMT* (A) and *Opn3* (C) genes in the E17 retina. Results of negative control experiments using sense probes are shown in panels **B** and **D**.
(TIF)

S4 Fig Western blot analysis of chicken TMT (A) and Opn3 (B) proteins. SDS-PAGE of protein samples (50 μg) derived from E19 chick heart, retina, and cerebellum. Proteins were transferred to polyvinylidene difluoride membrane and then stained with Coomassie Brilliant Blue to confirm that protein bands transferred fully to the membrane for western blot analysis. Slightly smaller chicken TMT-L protein was detected in the retina and cerebellum (~41 kDa) than in of chicken Opn3 protein is detected in the heart, retina, and cerebellum (~43 kDa). Antigen peptide-absorbed antibodies gave essentially no bands. Original blots with molecular size markers are shown in **C**. All blots are shown with molecular weight ladder in kDa.
(PDF)

S5 Fig Verification of antibody specificity. Antibody specificities were verified by incubating with antigen-absorbed anti-chicken Opn3- or anti-chicken TMT-antibodies in chicken retina and cerebellum. No specific labeling was detected with absorbed antibodies (pep [+]). Scale bars: 20 μm in **A-D** and 50 μm in **E-H**. Abbreviations: gcl, ganglion cell layer; ipl, inner plexiform layer; inl, inner nuclear layer. ml, molecular layer of the cerebellum; pl, Purkinje cell layer; gl, granule cell layer.
(TIF)

S6 Fig Large magnification of horizontal cells as shown in Fig 7F–7T. Left panels show localization of cTMT-L (green), middle panels show horizontal cell markers (magenta), and right panels show merged views.
(TIF)

S7 Fig Chicken TMT immunoreactive (IR) cells are not present in the paraventricular organ (PVO) of chick hypothalamus at P10. PVO is a photosensitive organ where Opn5m (a type of opsin 5) and serotonin are expressed in birds [5]. Left: Schematic diagram of chick brain through the posterior hypothalamus, showing the location of the PVO. Serotonin-IR cells (magenta) in the PVO are not positive for chicken TMT-L. Co, optic chiasm; IIIv, third ventricle. Scale bar: 50 μm.
(TIF)

S1 Table PCR primers used in this study. (XLSX)

S2 Table List of accession numbers of the genes whose sequences were used to construct Figs 1 and S1. (XLSX)

S3 Table Genes flanking the *opn3* locus shown in Figs 2 and 3. (XLSX)

S4 Table Antibodies used in IHC. (XLSX)
