# Supplementary material for: Two Opsin 3-Related Proteins in the Chicken Retina and Brain: A TMT-Type Opsin 3 Is a Blue-Light Sensor in Retinal Horizontal Cells, Hypothalamus, and Cerebellum
Source: PLoS One. 2016 Nov 18;11(11):e0163925. doi: 10.1371/journal.pone.0163925 (PMC5115664; doi:10.1371/journal.pone.0163925)
Supplement: S1 Fig — Chicken TMT opsin (cTMT) and chicken encephalopsin (cOpn3) are aligned with other opsin 3-related proteins. Bovine rhodopsin is used as reference for counterion position. (A) Transmembrane domain 3 (TM3), intracellular loop 2 (ICL2), TM4, and extracellular loop 2 (ECL2) are shown. E113, (D/E)R(Y/W) motif, and E181 are highlighted. (B) TM6, ECL3, TM7, and C terminal domains are shown. CWxP motif, K296, and NPxxY motif are highlighted. (PDF) [file pone.0163925.s001.pdf]

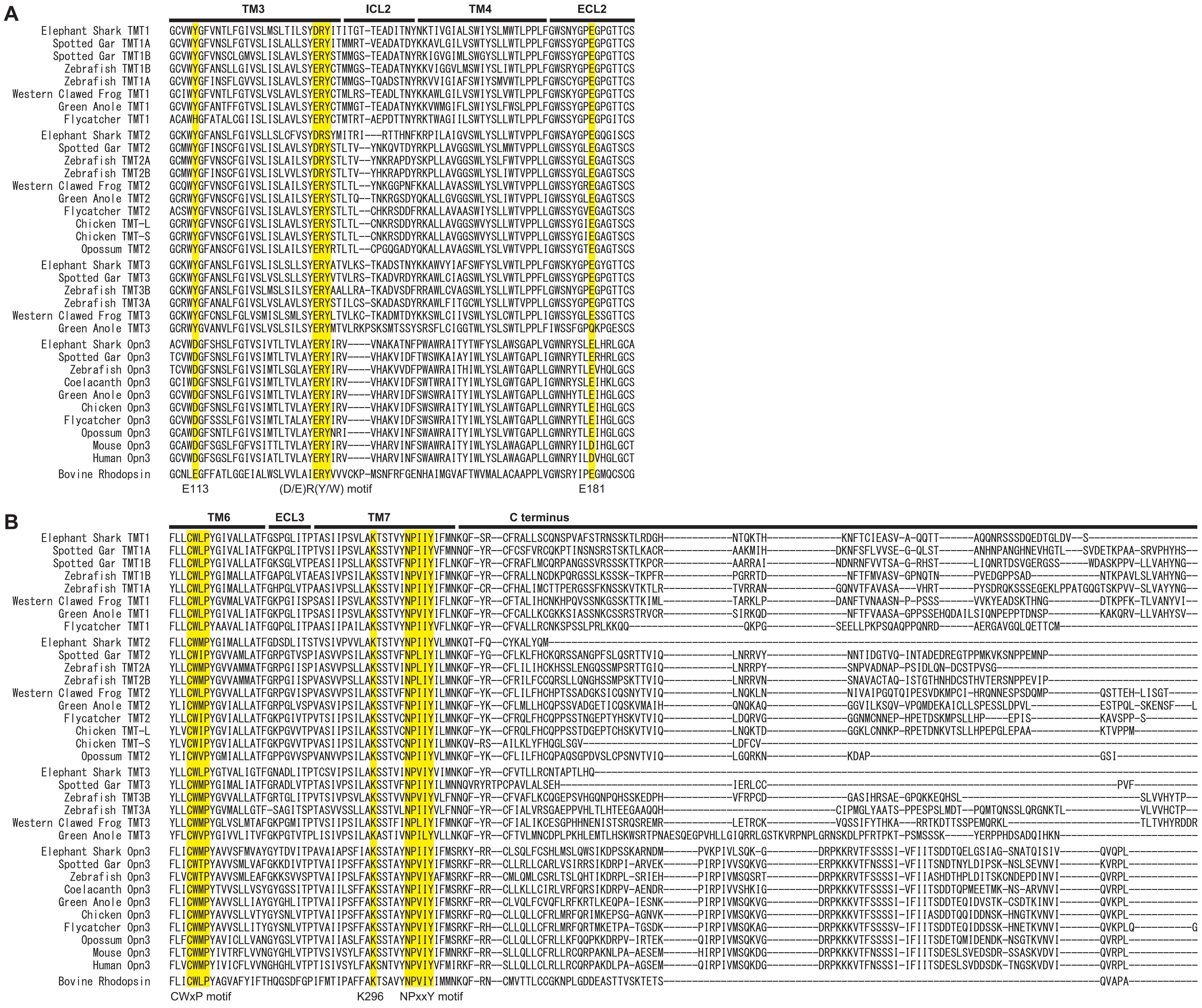
A
TM3
ICL2
TM4
ECL2
Elephant Shark TMT1
Spotted Gar TMT1A
Spotted Gar TMT1B
Zebrafish TMT1B
Zebrafish TMT1A
Western Clawed Frog TMT1
Green Anole TMT1
Flycatcher TMT1
Elephant Shark TMT2
Spotted Gar TMT2
Zebrafish TMT2A
Zebrafish TMT2B
Western Clawed Frog TMT2
Green Anole TMT2
Flycatcher TMT2
Chicken TMT-L
Chicken TMT-S
Opossum TMT2
Elephant Shark TMT3
Spotted Gar TMT3
Zebrafish TMT3B
Zebrafish TMT3A
Western Clawed Frog TMT3
Green Anole TMT3
Elephant Shark Opn3
Spotted Gar Opn3
Zebrafish Opn3
Coelacanth Opn3
Green Anole Opn3
Chicken Opn3
Flycatcher Opn3
Opossum Opn3
Mouse Opn3
Human Opn3
Bovine Rhodopsin
E113
(D/E)R(Y/W) motif
E181
B
TM6
ECL3
TM7
C terminus
Elephant Shark TMT1
Spotted Gar TMT1A
Spotted Gar TMT1B
Zebrafish TMT1B
Zebrafish TMT1A
Western Clawed Frog TMT1
Green Anole TMT1
Flycatcher TMT1
Elephant Shark TMT2
Spotted Gar TMT2
Zebrafish TMT2A
Zebrafish TMT2B
Western Clawed Frog TMT2
Green Anole TMT2
Flycatcher TMT2
Chicken TMT-L
Chicken TMT-S
Opossum TMT2
Elephant Shark TMT3
Spotted Gar TMT3
Zebrafish TMT3B
Zebrafish TMT3A
Western Clawed Frog TMT3
Green Anole TMT3
Elephant Shark Opn3
Spotted Gar Opn3
Zebrafish Opn3
Coelacanth Opn3
Green Anole Opn3
Chicken Opn3
Flycatcher Opn3
Opossum Opn3
Mouse Opn3
Human Opn3
Bovine Rhodopsin
CWxP motif
K296
NPxxY motif
