# Supplementary figures and images for: Two Opsin 3-Related Proteins in the Chicken Retina and Brain: A TMT-Type Opsin 3 Is a Blue-Light Sensor in Retinal Horizontal Cells, Hypothalamus, and Cerebellum
Source: PLoS One. 2016 Nov 18;11(11):e0163925. doi: 10.1371/journal.pone.0163925 (PMC5115664; doi:10.1371/journal.pone.0163925)

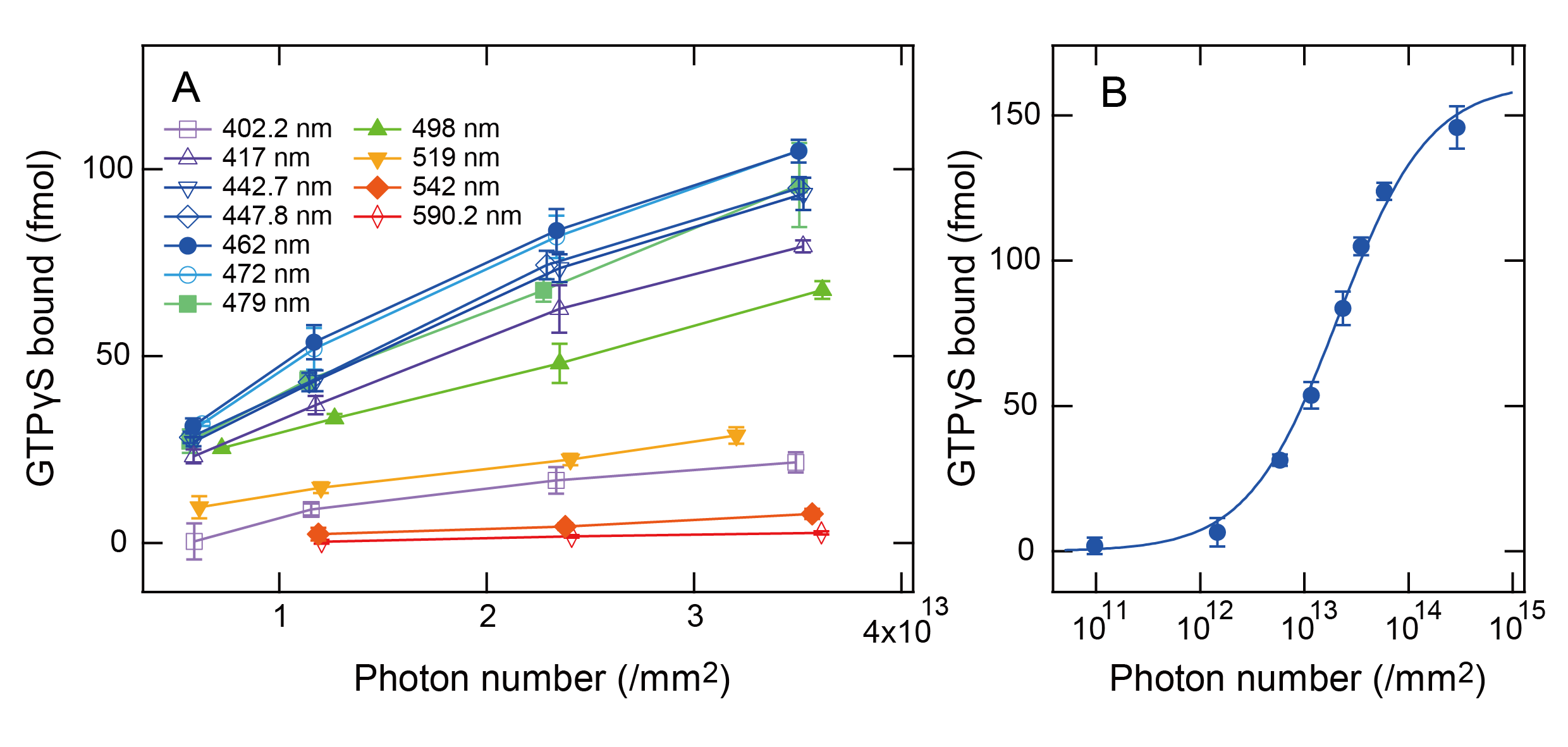

Supplement: S2 Fig — (A) Go activation efficiencies of cTMT-L in HEK293T cell membranes were measured by irradiation of selected wavelength light in the region of light intensity where linear relationships between Go activation efficiency and light intensity were mostly observed. The measurements were performed with lights of eleven different wavelengths at 0°C. The plotted data were calculated by subtracting the activity without light irradiation from that measured with irradiation within each light conditions. (B) Go activation profile of cTMT-L in HEK293T cell membranes following irradiation with 462 nm-light of different intensities. The data were fitted with a Hill equation: y = Basal + (Max − Basal) / (1 + EC50 / x), (Hill coefficient = 1, solid curve) and the EC50 value was calculated to be 2.12 × 10^13^ photons/mm2. (TIF) [file pone.0163925.s002.tif]

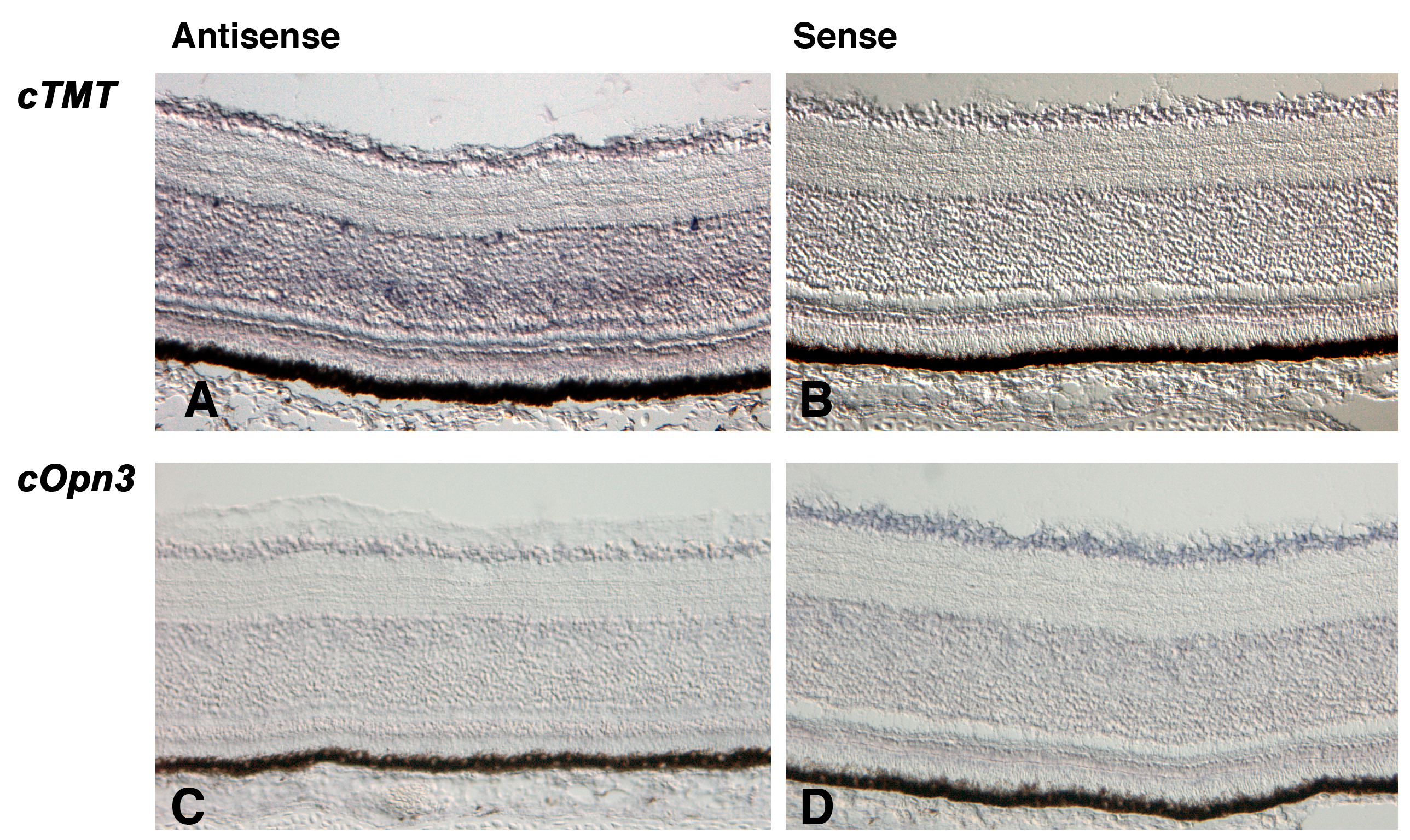

Supplement: S3 Fig — Results of negative control experiments using sense probes are shown in panels B and D. (TIF) [file pone.0163925.s003.tif]

**A**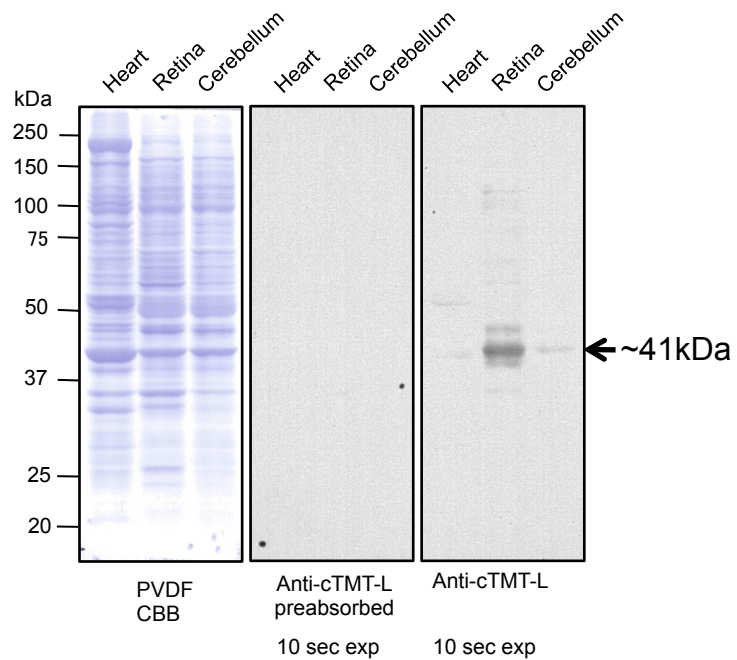**B**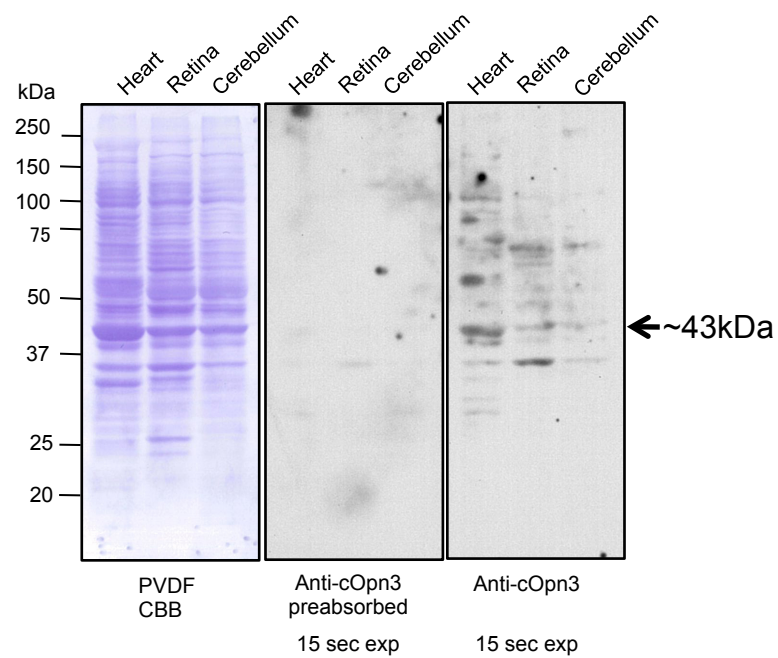**C**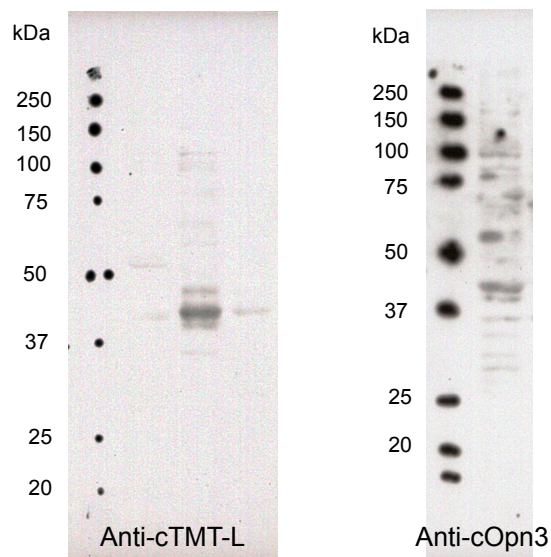

Supplement: S4 Fig — SDS-PAGE of protein samples (50 μg) derived from E19 chick heart, retina, and cerebellum. Proteins were transferred to polyvinylidene difluoride membrane and then stained with Coomassie Brilliant Blue to confirm that protein bands transferred fully to the membrane for western blot analysis. Slightly smaller chicken TMT-L protein was detected in the retina and cerebellum (~41 kDa) than in of chicken Opn3 protein is detected in the heart, retina, and cerebellum (~43 kDa). Antigen peptide-absorbed antibodies gave essentially no bands. Original blots with molecular size markers are shown in C. All blots are shown with molecular weight ladder in kDa. (PDF) [file pone.0163925.s004.pdf]

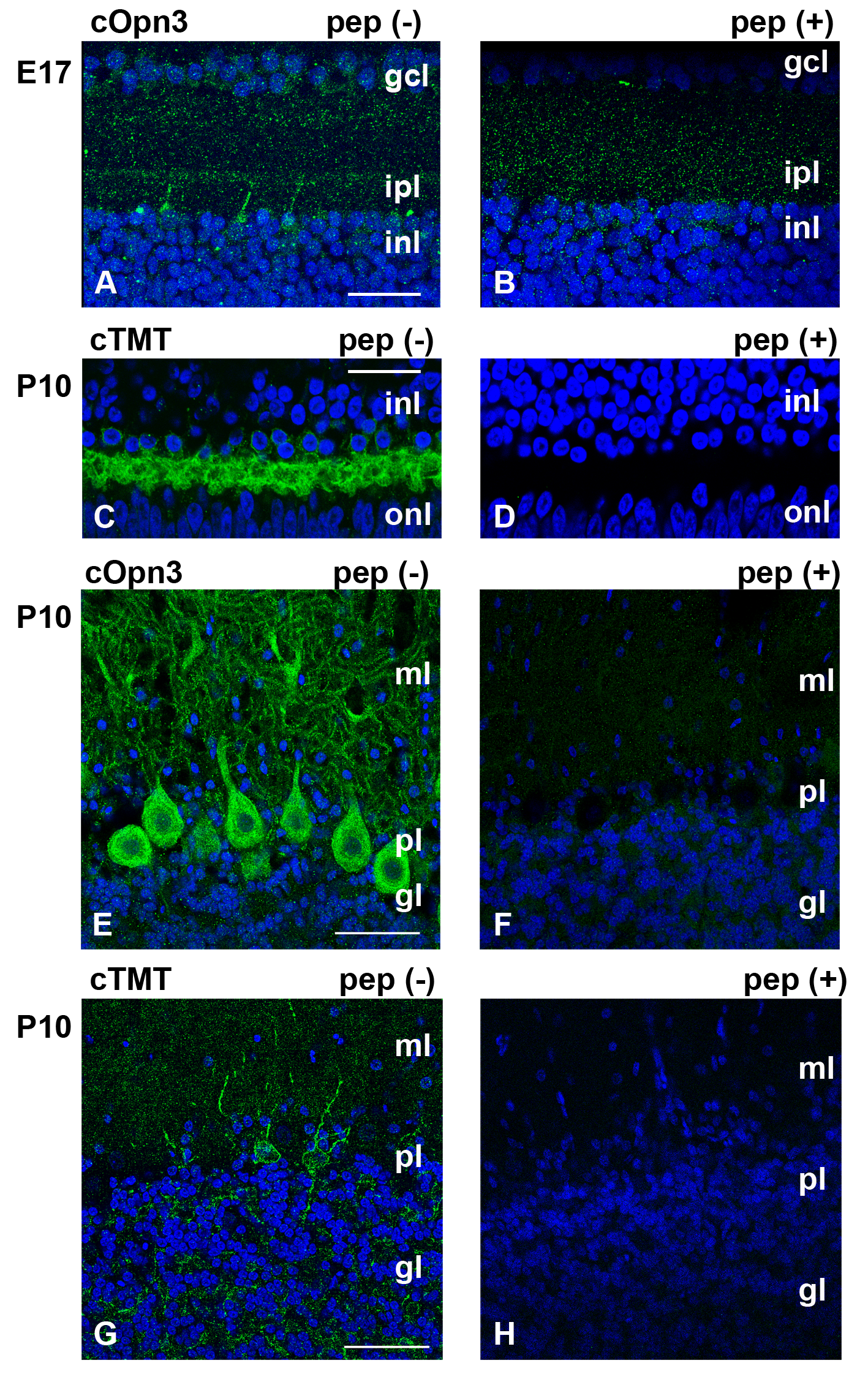

Supplement: S5 Fig — Antibody specificities were verified by incubating with antigen-absorbed anti-chicken Opn3- or anti-chicken TMT-antibodies in chicken retina and cerebellum. No specific labeling was detected with absorbed antibodies (pep [+]). Scale bars: 20 μm in A-D and 50 μm in E-H. Abbreviations: gcl, ganglion cell layer; ipl, inner plexiform layer; inl, inner nuclear layer. ml, molecular layer of the cerebellum; pl, Purkinje cell layer; gl, granule cell layer. (TIF) [file pone.0163925.s005.tif]

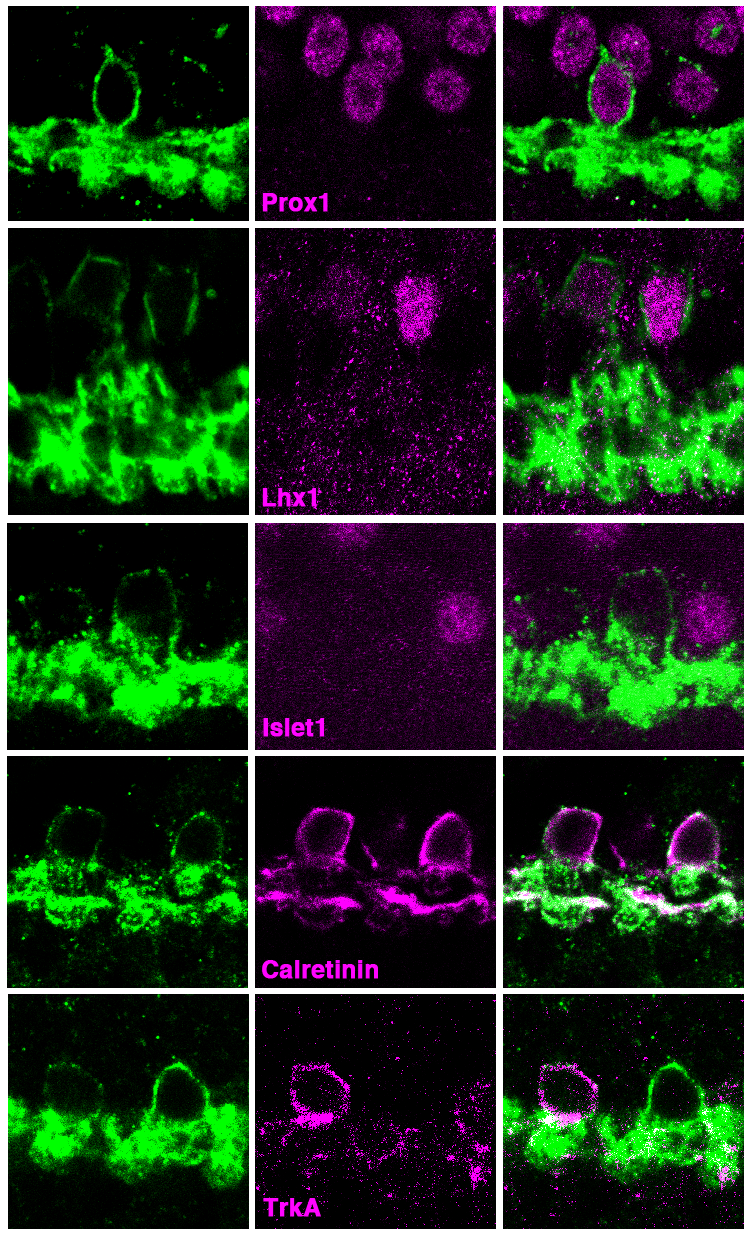

Supplement: S6 Fig — Left panels show localization of cTMT-L (green), middle panels show horizontal cell markers (magenta), and right panels show merged views. (TIF) [file pone.0163925.s006.tif]

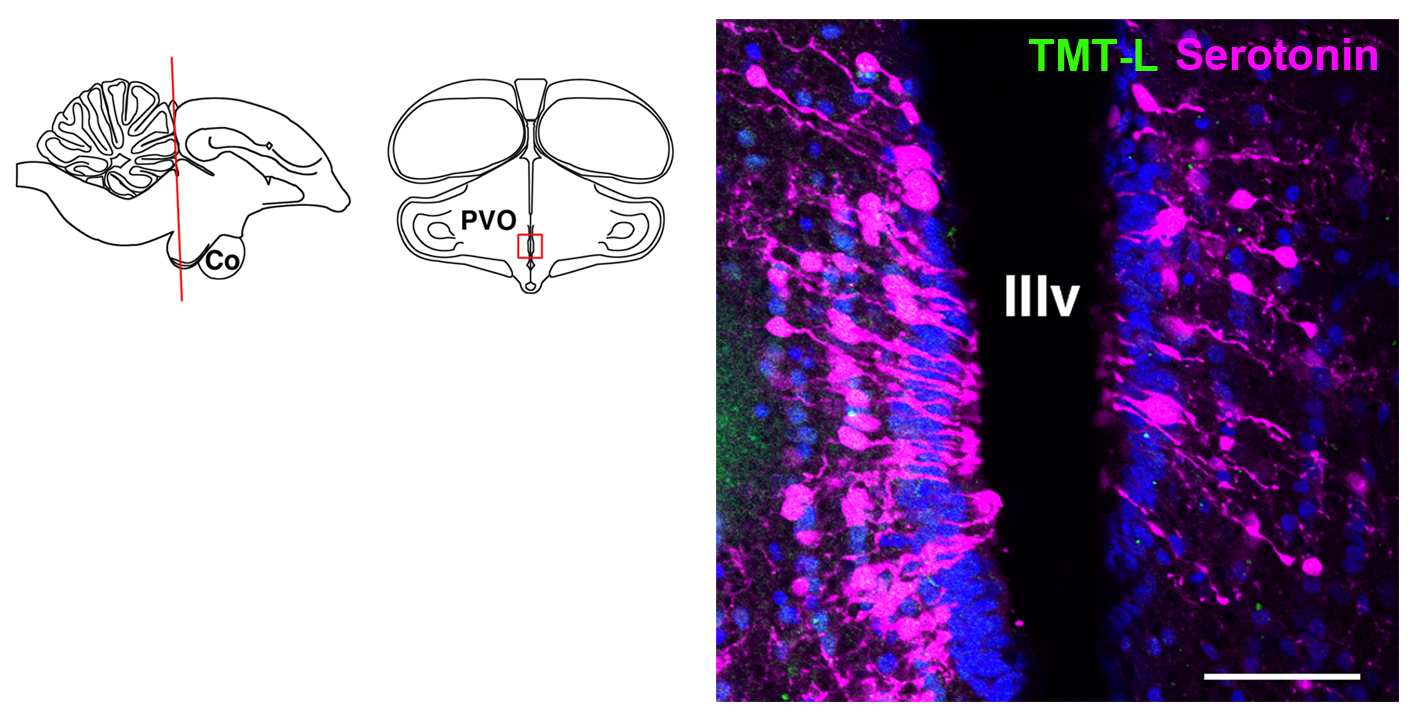

Supplement: S7 Fig — PVO is a photosensitive organ where Opn5m (a type of opsin 5) and serotonin are expressed in birds [5]. Left: Schematic diagram of chick brain through the posterior hypothalamus, showing the location of the PVO. Serotonin-IR cells (magenta) in the PVO are not positive for chicken TMT-L. Co, optic chiasm; IIIv, third ventricle. Scale bar: 50 μm. (TIF) [file pone.0163925.s007.tif]
